# Poly(Vinyl Acetate) Paints: A Literature Review of Material Properties, Ageing Characteristics, and Conservation Challenges

**DOI:** 10.3390/polym15224348

**Published:** 2023-11-07

**Authors:** Morana Novak, Bronwyn Ormsby

**Affiliations:** Tate Britain, Millbank, London SW1P 4RG, UK; bronwyn.ormsby@tate.org.uk

**Keywords:** poly(vinyl acetate), vinyl, modern paints, paint properties, degradation, cleaning

## Abstract

Since their development in the 1950s, poly(vinyl acetate) [PVAc] paints (also known as vinyl) have been used by many artists, most notably in countries such as Spain, Portugal, and the United Kingdom; they are also used globally as a common binder for house paints. However, only a relatively limited number of heritage scientific studies have focused on vinyl paints. Consequently, many critical aspects of this material, such as the degradation processes, variations in paint formulations, and responses to conservation treatments, remain largely understudied. This article aims to summarise the available relevant information on poly(vinyl acetate) paints from both the scientific and the conservation practice perspectives. The article provides a brief overview of the development of poly(vinyl acetate) paints as artist-grade and household products and the known differences in their formulations. It also focuses on poly(vinyl acetate) ageing behaviour, the physicochemical properties, the recent scientific research on poly(vinyl acetate) material characterisation and degradation, and the main conservation issues regarding these paints, such as those relating to cleaning treatments.

## 1. Introduction

The first half of the 20th century witnessed major developments in the field of synthetic paints, which have found a wide range of applications in everyday life [1,2]. In addition to their extensive use as household paints, many products have been developed as artist-grade paints and related materials [2,3]. The advent of these types of modern polymeric materials facilitated changes in artistic expression in new and innovative ways, which were not as possible with traditional oil paint materials. These new paints also provided several advantages over traditional paints, such as higher resistance to light ageing, improved flexibility, easier preparation and handling, minimal use of organic solvents in some cases, and rapid drying.

Although many synthetic materials had been introduced as potential binding media for paints, only a few types dominated the market. The key 20th-century synthetic polymer binders included acrylics, poly(vinyl acetate), oil-modified alkyds, and nitrocellulose, respectively [1]. Most 20th-century artist products have been dominated by acrylic emulsion binders or modern oil formulations [1,2,4,5,6]. At the same time, however, poly(vinyl acetate) paints were and continue to be used by artists and have been praised by some artists for their properties [2]. Although poly(vinyl acetate) paints were never as popular as acrylic emulsion paints, which came to the market around the same time through to the 1970s, both household and artist-quality poly(vinyl acetate) paints were used by many notable artists. Some of these include Bridget Riley, Sidney Nolan, Kenneth Noland, Fred Graham, Nelson Kenny, Colin McCahon, Ralph Hotere, Theo Schoon, Gordon Walters, Harry Wong, Ian Fairweather, Joaquim Rodrigo, Ângelo de Sousa, Julião Sarmento, Walter de Maria, Agostino Bonalumi, Toti Scialoja, and Gastone Novelli [1,2,7,8,9,10,11,12]. Although they are based on the same vinyl binder, the difference between the household paints and the artist-quality paints is that the latter are generally of higher quality, due to the higher content of pigments and thickeners and lower amount of extenders [13,14]. Although commercial artist-quality poly(vinyl acetate) products were available, research has shown that some of the previously mentioned artists employed industrial and household paints within their works [1,3,9,15,16,17]. In addition, some artists prepared their own paints by mixing pigments with commercial poly(vinyl acetate) resins and glues (such as wood glue) or by adding pigments to household paints in order to obtain the desired colour hue; these artists include Bridget Riley, Alberto Burri, Sidney Nolan, Ian Fairweather, Joaquim Rodrigo, and Julião Sarmento.

**Figure 1:**
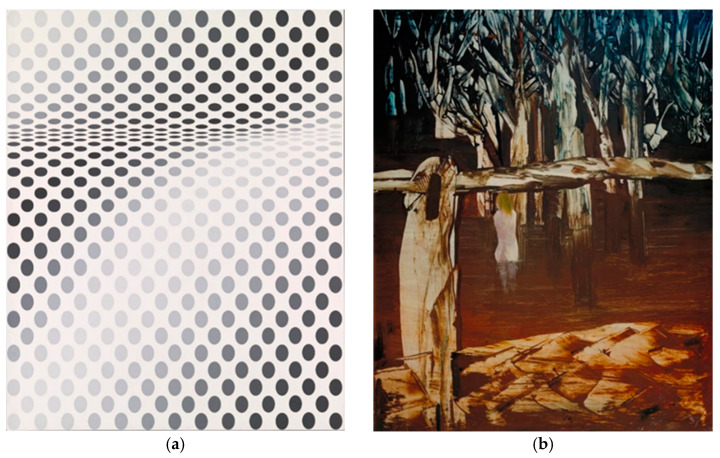
Paintings including the use of poly(vinyl acetate) paints: (**a**) Bridget Riley, *Hesitate*, 1964. Emulsion on board, 106.7 × 112.4 cm. London, Tate, T04132, © Bridget Riley, Photo: Tate, London, UK; and (**b**) Sidney Nolan, *Women and Billabong*, 1957. Poly(vinyl acetate) paint on hardboard, 152.4 × 121.9 cm. London, Tate, T00151, © Sidney Nolan Trust, Presteigne, UK; all rights reserved 2023, Bridgeman Images, London, UK, Photo: Tate, London, UK. NB: images **not** subject to creative commons license.

Two examples of poly(vinyl acetate) paintings in the Tate collection, *Hesitate* by Bridget Riley and *Women and Billabong* by Sidney Nolan, are presented in Figure 1. Bridget Riley’s painting forms the focus of a current research project (GREen ENdeavor in Art ResToration (GREENART), https://www.greenart-project.eu/, accessed on 2 November 2023. As part of this project, Tate leads on Work Package 2, Task 2.3 ‘Assessment of green cleaning fluids and green gels’, which assesses the green solutions produced through GREENART: https://www.tate.org.uk/about-us/projects/greenart, accessed on 2 November 2023). 

In addition to artists mixing their paints, the presence of different binding media in artworks is common due to artists mixing and/or using different types of paint media. One example is Mario Schifano, whose *Incidente D662* was primarily executed using an (oil-modified) alkyd polymer, with vinyl paint used for the orange colour [10]. In *Potrebbe Uscire*, Gastone Novelli used gypsum for the white areas and black vinyl-based paint for the darker areas [11]. On occasion, the presence of other binding materials is also a consequence of past conservation treatment [11]. The analyses of two vinyl paintings from Ângelo de Sousa (untitled work, inventory numbers AS/p036 and AS/p159) also indicated the presence of an acrylic varnish layer [18]. Some Sidney Nolan paintings, such as *Peter Grimes’s Apprentice*, were made using both oil and poly(vinyl acetate) paints [15,19]. At present, the effects of conservation treatment, as well as the general and specific ageing behaviours of these types of mixed media artworks, remain largely unexplored [15].

The use of different supports creates an additional challenge for the conservation of artworks, as some supports, such as cotton canvases for example, can induce colour change in modern paints [9,20]. Bridget Riley’s supports for her works in Tate’s collection range from hardboard (*Hesitate* and *Fall*) to a large collection of paper prints, prints on Perspex (acrylic) sheets [21], and numerous artworks made of poly(vinyl acetate) on canvas, such as *Deny 2* and *Late Morning*. Many other artists also use a range of supports. Sidney Nolan also used paper, hardboard, and canvas supports [15]. Ângelo de Sousa also used hardboard, paper, and paperboard glued on Platex board as supports for his works [8,13,18].

Two of the main advantages of vinyl paints were that they were relatively inexpensive and offered properties comparable to those of the more expensive acrylic paints [1,2]. One of the disadvantages was, however, that some of the household brands were only available in a limited colour range; therefore, the artists often added their own pigments [1,3]. In addition, it is stated that vinyl emulsion paints are more prone to weathering compared to acrylics [5,18], due to their weaker binding power and mechanical strength.

Regardless of their popularity, modern paints, including poly(vinyl acetate) present their own set of challenges, such as inherent material instability, complex chemical composition, and relatively little research into the effects of cleaning and on devising optimal methods for conservation treatment. Many of these (unvarnished and unprotected) artworks currently require remedial treatments—primarily due to the accumulation of dust, grime, scuffs, and other marks over several decades. This provides additional motivation to further characterise the material properties of these paints to help inform conservation treatment and preservation decisions. The conservation-related aspects of some modern paints have been in the research spotlight for the last few decades; thus far, this has enabled key changes to conservation practices through the building of a significant body of research. As stated earlier, much of this effort has focused on acrylic emulsion and oil paints; however, for poly(vinyl acetate)-based paints, most of their material properties and degradation processes are not as thoroughly understood.

Of the available literature, the publications that have focused on poly(vinyl acetate) paints to date range from studies on the artist’s use of these paints to variations in paint formulations, ageing behaviour, methodological approaches to characterising and analysing poly(vinyl acetate) paints, and the effects of different solvent-based conservation treatments (such as cleaning) on works of art [13,18,22]. This review paper presents the most relevant published information on poly(vinyl acetate) paint materials to help inform subsequent research and to provide information on the risks related to the surface cleaning treatment of poly(vinyl acetate) paint or poly(vinyl acetate)-painted works of art. The review is divided into several sections, such as those on the historical development and formulation of poly(vinyl acetate) paints, physical properties, ageing behaviour, and material analysis, as well as any relevant conservation and preservation research.

## 2. History and Formulation

**Figure 2:**
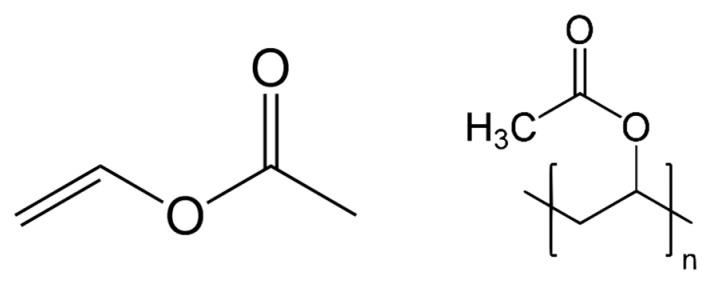
Chemical structures of vinyl acetate and poly(vinyl acetate).

Poly(vinyl acetate) was first synthesised by Fritz Klatte in 1912 through the polymerisation of vinyl chloroacetate monomer units [23,24]. Vinyl monomers are generally produced via the reaction of acetic acid and acetylene in the presence of mercury salts, which act as reaction catalysts. Because of the presence of double (–C=C–) bonds in their structure, the monomer units of vinyl acetate polymerise with each other and form long chains of poly(vinyl acetate), in which the carbon atoms are linked through single covalent bonds (–C–C–), as shown in Figure 2 [25,26].

The properties of the final polymer product depend on the reaction conditions (temperature), initiators and catalysts, type of solvents used, and their concentration [27]. At first, poly(vinyl acetate) was used as an adhesive, and two decades later, in the 1930s, it was introduced to the market as a waterborne resin [1]. It is currently reported that the first application of poly(vinyl acetate) in the conservation field was in the early 1930s, when it was used as a consolidation material for wall paintings [28]. As a paint material, poly(vinyl acetate) was first used in the form of a solution, which was prepared by dissolving the resin in a selected organic solvent [1]. However, poly(vinyl acetate) in its solution form was of limited use in the artistic sense, and it was soon replaced by waterborne emulsions, in which poly(vinyl acetate) was dispersed in water. In this form, poly(vinyl acetate) paints became increasingly popular during the 1950s. The first commercial poly(vinyl acetate) paints, including artist-grade lines, were introduced in the 1950s [1,3,29,30].

**Figure 3:**
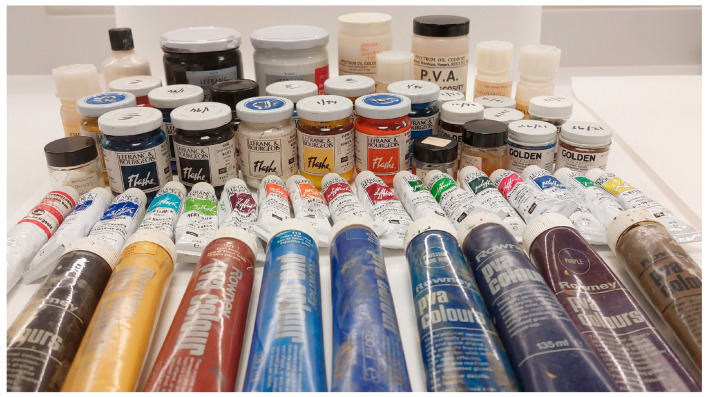
Poly(vinyl acetate) artists’ paints and resins. Photo: Morana Novak, Tate.

In general, modern paints, including poly(vinyl acetate), fall into two separate categories: artist-quality products and house paints [2]. Starting in the 1950s, poly(vinyl acetate) paints were mainly produced as household paints. The first known artist emulsion poly(vinyl acetate) paint, Polymer Tempera, was produced in the 1940s by the company Borden Co. (Columbus, OH, USA) [1]. The companies that led the production of artists’ paints in the first few years were Rowney (Bracknell, UK), Lefranc and Bourgeois (Le Mans, France), and Spectrum Oil Colours (Cwmbran, UK). The first artist-quality poly(vinyl acetate) products became available in the mid-1960s in the UK and were manufactured by Rowney [15]. Rowney marketed two types of poly(vinyl acetate) in the form of premixed colours, as well as pure poly(vinyl acetate) homopolymer emulsion, to which pigments could be mixed. Soon after, aqueous poly(vinyl acetate) homopolymer emulsions were developed by Spectrum Oil Colours. In addition, Spectrum offered a range of products with various mixtures of acryl and vinyl emulsions. In the late 1950s and early 1960s, Derivan (Rhodes, New South Wales, Australia), Taubmans (Clayton, Victoria, Australia), British Paints Ltd. (Clayton, Victoria, Australia), and BALM Paints Pty Ltd. (Melbourne, Victoria, Australia) produced a range of poly(vinyl acetate) and acrylic paint media [7,17,31]. In Portugal in the 1950s, Robbialac (Vila Real, Portugal) released a range of vinyl emulsion paints under the commercial name of Robbialac Emulsion Paint (REP), and A Favrel Lisbonense (Lisbon, Portugal) produced artist-quality vinyl products, namely Sabu and Geo fluorescence paints and Vulcano V7 vinyl glue [32]. New Masters (Hague, The Netherlands) produced a range of paints based on vinyl–acrylic copolymers, and Golden Artist Colors (New Berlin, NY, USA) has produced conservation-grade poly(vinyl acetate) paints which are dissolved in alcohol [33,34]. Lefranc and Bourgeois started producing their range of poly(vinyl acetate) paints in the late 1950s; these paints were copolymerised with either vinyl versatate (VeoVa) copolymers or acrylates [2]. Versatates are highly branched esters made of C9 and C10 vinyl monomers [35]. Vinyl-VeoVa paints have been sold under the commercial name Flashe, and vinyl acrylates have been sold under the name Polyflashe. Another French manufacturer with poly(vinyl acetate) paints in their range was Ripolin (Paris, France) [1]; Reeves Polymers (London, UK) also manufactured a blend of vinyl acrylic paints [36]. Some of these historic poly(vinyl acetate) paint products are presented in Figure 3. With the exception of the poly(vinyl acetate) paints by Lefranc and Bourgeois and Golden Artist Colors (marketed as Conservation paints) [33], which are still available today, most of the other manufacturers discontinued their poly(vinyl acetate) artists’ paint lines several decades ago [36].

At present, the main uses of poly(vinyl acetate) are interior house paints, and their main markets are based in the UK and Europe. In addition to being mostly known as a binding medium for modern paints, poly(vinyl acetate) compounds have several other applications in conservation, mainly as cleaning gels, coatings, varnishes, and retouching and adhesive materials [34,37,38,39,40,41]. Outside of conservation, polyvinyl acetate has been used as a composite material for various applications, such as adhesion, construction and medical materials [42,43,44,45]. In art conservation, there are several reported applications of poly(vinyl acetate) composite materials, and they are used for conservation, mostly as cleaning materials. Angelova et al. and Al-Emam et al. synthetised and characterised composites made of partially hydrolysed poly(vinyl acetate) cross-linked with borate and agarose, which, due to their rheological and structural properties, have the potential to be used in heritage, as cleaning gels for example [46,47,48,49]. Recently, Ricciotti et al. also proposed the use of novel geopolymer and poly(vinyl acetate) composites as adhesives for the interventive conservation of various heritage materials [50].

## 3. Physical Properties

Poly(vinyl acetate) is a colourless homopolymer comprising large polymer chains of vinyl acetate units, as presented in Figure 2 [37]. The main component of poly(vinyl acetate) paints, in both their solution and emulsion forms, is poly(vinyl acetate) resin, which is most often produced separately and then used for paint manufacturing. Pure poly(vinyl acetate) resin is mixed with other components, such as pigments and additives, in order to make the final product. For solution paints, organic solvents are used, while for emulsion paints water is used as the key diluent.

Pure poly(vinyl acetate) resin is a clear and colourless liquid which is insoluble in water, aliphatic hydrocarbons, waxes, and fats, but it is soluble in some organic solvents, such as ketones, esters, alcohols, and aromatic hydrocarbons [30]. The polymer in its pure form does not have robust inherent mechanical properties and flexibility [51]. As with acrylics, poly(vinyl acetate) is a thermoplastic material, meaning that ambient temperature influences physical properties such as softness and flexibility. Therefore, a dried poly(vinyl acetate) film is relatively brittle due to its glass transition temperature (*T_g_*) at approximately 30 °C, depending on the molecular weight of the vinyl polymer [38,52]. Consequently, most commercial solvent-borne and water-borne poly(vinyl acetate) products contain additives such as external (non-bonded) plasticisers to improve their flexibility during film formation and beyond. The first poly(vinyl acetate) formulations used external plasticisers, most often phthalates, such as dibutyl phthalate, diisobutyl phthalate, bis(2-ethylhexyl) phthalate, but also benzoates and triphenyl phosphate, which also acts as a flame retardant (Table 1) [5,51,53,54].

**Figure 4:**
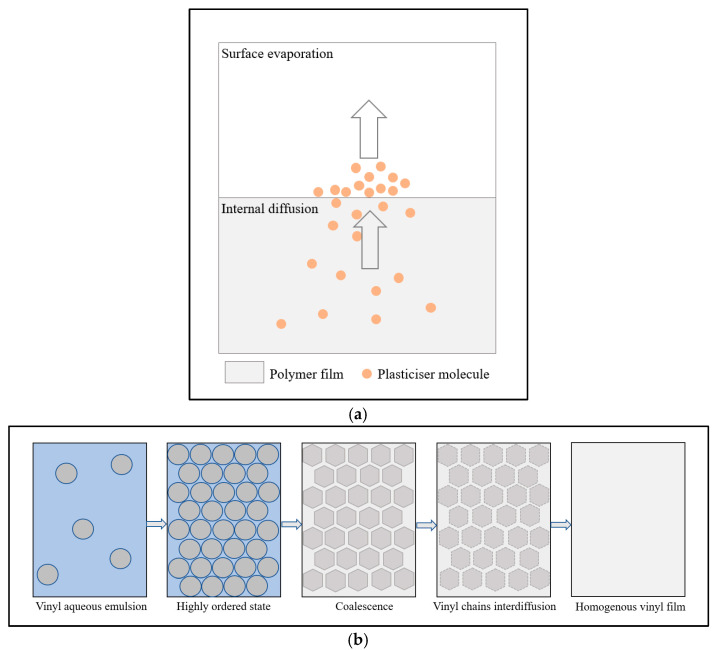
Some physical processes encountered in poly(vinyl acetate) films: (**a**) plasticiser migration and loss (the arrows indicate the direction of plasticiser migration) and (**b**) film drying and formation. (**b**) is adapted with permission from Zumbühl et al. [62]. © 2007 J. Paul Getty Trust.

One of the inherent instabilities of poly(vinyl acetate) paints is plasticiser loss. This process occurs through internal diffusion and the surface evaporation of external plasticisers, as these plasticiser molecules are not linked to the polymer chain through primary chemical bonds [55]. The loss of external plasticisers leaves paint films brittle and can result in plasticiser deposits on the paint surface, causing the films to become tacky and thereby enhancing dirt and dust build-up and retention [56,57,58]. Similarly, surfactants can migrate and deposit on the paint surfaces [59]. A simplified diagram of the process of plasticiser migration and loss is shown in Figure 4a. Consequently, external plasticisation was replaced by the use of internal plasticisers, which resulted in key paint formulation changes in the 1960s. Internal plasticisation was achieved through mixing poly(vinyl acetate) with softer monomers, such as acrylates (*n*-butyl acrylate [nBA] and 2-ethyl hexyl acrylate [2-EHA]) or vinyl versatates [2,3], which are highly branched esters made of C9 and C10 monomer vinyl units [35]. Due to their lower glass transition temperatures, −50 °C for 2-EHA acrylates and −3 °C for versatates, the addition of these compounds imparts flexibility to the vinyl homopolymer film [36,60]. Copolymers of vinyl with versatates and acrylates also tend to be more resistant to ultraviolet (UV) ageing and to the action of solvents, such as water, as well as acids and alkalis. As a result, these formulations rapidly replaced external plasticised resins and paints [30,51]. However, some modern vinyl paint formulations remain plasticised and use both external and internal plasticisers [61].

As with the other polymers used as binding media in art, such as acrylics, the solution and emulsion forms of poly(vinyl acetate) paints exhibit different properties [1,36]. The first difference is the interaction between the polymer and the solvent medium and the associated drying mechanism. In the solution form, polymer chains are dissolved in a solvent, which is most often an organic solvent, such as an alcohol. A vinyl solution paint of conservation-grade is being produced by the aforementioned Golden Artist Colors. A paint film is formed from the solution through solvent evaporation. This creates a dry film that can be re-dissolved in the same solvent [2,34]. Poly(vinyl acetate) solution paints are often touch dry after a few hours. In contrast, for the emulsion forms, the polymers are dispersed in water as spherical particles where various surfactants are added to stabilise the emulsions. Emulsion paints have several advantages over solution products, such as low emissions of volatile organic compounds. Furthermore, the use of water makes them more environmentally friendly; in addition, emulsions are modifiable and can be produced as versatile products with varied properties [27]. The film-drying process of emulsions and emulsion paints is complex, multi-phased, and known as coalescence (Figure 4b). Initially, the rate of the film-drying process is entirely controlled by water evaporation. The polymer molecules move closer together through Brownian motion in parallel with water evaporation [63]. In the second phase, as water evaporation progresses further, the polymer molecules become even more concentrated, stay in close contact, and start to deform, coalesce, and entangle together to form a solid film. The remaining water diffuses through the polymer matrix to the surface, which is a much slower process than the initial water evaporation step. This marks the final step of the drying process [1,62,64,65]. However, because both acrylic and poly(vinyl acetate) waterborne (emulsion) paints contain hydrophilic additives, some water remains in the system even after coalescence. Owing to the presence of water, added plasticisers, and hydrophilic additives, the polymer particles can create small channels between them during the drying process. Through these channels, the remaining water and other materials can move in and out of the film [66], which is likely to form the key mechanism behind the swelling of dried paint films with water and other polar solvents.

The drying process can last for longer periods, such as several years after the initial film application, and several researchers noted a weight change in touch dry emulsion paint films, including poly(vinyl acetate), over time [51,67]. This was explained through the loss of residual water and slower evaporating additives, such as a coalescing solvent, which is usually a glycol-type solvent added to temporarily lower the film glass transition temperature during the early stages of drying.

Different mass-transfer models describe the drying process of various emulsion paints [68,69,70]. The potential to study the film formation mechanism in polymer emulsion paints, using analytical techniques such as dynamic light scattering, has mostly been employed for acrylics, styrene, and styrene–acrylic aqueous emulsions [71,72,73,74,75]. Ferreira et al. reported using this technique to monitor the photodegradation of poly(vinyl acetate) resins and paints to determine particle size and to explore whether this has any influence on the cross-linking of polymer chains [13,16]. This method is used to measure particle size distribution during polymerisation and film drying, as well as the interactions between polymers and other particles present in emulsions, such as inorganic pigments (TiO_2_) and surfactants [72,76]. This technique measures the rate of Brownian motion of the particles present in the emulsion using a monochromatic light source, where changes in the intensity of the light scattered from these particles are monitored [77], providing information on paint coalescence and the film formation process, as well as the ongoing stability of emulsions.

As with the solutions, poly(vinyl acetate) emulsions become touch dry in a very short time; however, they cannot be re-dissolved in water after drying (as with the acrylic emulsion paints). The solution and emulsion as unpigmented media also differ in their visual appearance, where the unpigmented poly(vinyl acetate) solution is transparent and the unpigmented emulsions are, unsurprisingly, more opaque [2]. Furthermore, the solution forms of paints exhibit a lower degree of polymerisation and molecular weights at 50,000–100,000, compared to the emulsion paints with molecular weights which are sometimes over 1,000,000 [5,78,79,80]. This in turn influences some of the dry film physical properties; e.g., vinyl paints produced through emulsion polymerisation exhibit improved mechanical properties due to higher chain entanglement and higher viscosity due to lower molecular mobility [81]. In addition, the glass transition temperature is higher for emulsion paints compared to the solution paints, due to their higher molecular weight [82].

## 4. Additives

As mentioned, poly(vinyl acetate) waterborne emulsion paints are complex mixtures of vinyl binders, pigments, fillers, extenders, and other additives [2,27,61,83]. These can be added during the synthesis of poly(vinyl acetate) resins or paint production. The main types of additives used in poly(vinyl acetate) paint formulations are identical to those used in acrylic emulsion paints, such as surfactants, antifoam agents, thickeners, coalescing agents, pH buffers, biocides, wetting agents, pigment dispersants, and freeze–thaw agents. The additives are used in order to modify the working properties of wet paints as well as the physical properties of the final product by influencing processes such as film formation. Furthermore, certain additives can play more than one role in a paint formulation; for example, cellulose derivatives are used as both thickeners and protective colloids, i.e., they improve polymer solubility, influence rheology, and sterically stabilise poly(vinyl acetate) paints. The main types of additives present in modern paints, including vinyl paints, as well as their roles and examples of compounds typically used, are listed in Table 1. Even if present at low concentrations, generally below 5%, the additives in vinyl paints can influence the final properties of the product, as well as ageing behaviour, physical properties, and vulnerability to conservation treatments such as cleaning [2,51]. Thus far, relatively little research has been conducted on the effects of various additives on the properties of poly(vinyl acetate) paints. The majority of the published studies have focused on external plasticiser loss in aged vinyl paint and resin films and the consequent deterioration of the mechanical properties of the films [61,84,85]. A few other researchers have also investigated the effects of simulated wet surface treatments on the leaching of plasticisers and surfactants [9,22,58].

**Table 1:** The list of additives present in the emulsion paints.

Additive	Role in the Emulsion Paints	Chemical Compounds Added to the Emulsion Paints [5,9,51,86,87,88,89,90,91]
Antifoam agent	Prevents the development of air bubbles during paint handling	Mineral and silicone oils
		Octanol
		Polydimethylsiloxane
Biocide	Prevents biological contamination of the paints during storage	Tin oxide
		Zinc oxide
		Mercury-based compounds
		Acrylamide
		2-n-octyl-4-isothiazolin-3-one
Coalescing agent	Improves the coalescing process between polymer molecules during the drying phase	Ester alcohols
		Benzoate esters
		Glycols
		Glycol ethers
		N-methyl-2-pyrrolidone
Fillers	Reduces the cost of paint production and improves the handling of the paint	Calcium carbonate
		Hydrated magnesium silicate
Freeze–thaw agent	Prevents the freezing of the paints when exposed to cold temperatures	Ethylene glycol
		Propylene glycol
pH buffer	Modifies paint pH to make it optimal for all the paint components (optimal between pH 8 and 10)	Ammonia
Pigment dispersant	Improves the dispersion of the solid pigment particles	Oligophosphates (calcium and potassium salts)
		Polyacrylic acids (sodium and ammonium salts)
Plasticisers	Improves the flexibility of the homopolymer	*External*
		Dibutyl phthalate
		Diethyl phthalate
		Isobutyl phthalate
		Bis (2-Ethylhexyl) phthalate
		Dipropylene glycol dibenzoate
		Diethylene glycol dibenzoate
		Triphenyl phosphate
		*Internal*
		Vinyl versatates
		N-butyl acrylate
		2-ethyl hexyl acrylate
Protective colloids	Improves the polymer solubility and sterically stabilises emulsion paints	Hydroxyethyl cellulose
		Methyl cellulose
		Poly(vinyl alcohol)
Surfactants	Disperses polymer and pigment molecules in water and electrostatically stabilises emulsion paint	Ethoxylated alkyl alcohols and phenols
		Alkyl sulphonates and sulphates
		Ethoxylated sulphonates and sulphates
		Phosphates
Thickener	Increases the paint viscosity (thicker paint) and improves the paint’s workability	Hydroxyethyl cellulose
		Methylcellulose
		Hydrophobically modified carboxymethylcellulose
		Hydrophobically modified ethoxylated urethane
		Polysaccharides (xanthan and guar gums)
Wetting agent	Reduces the surface tension around the pigment particles and increases their wettability	Alkyl phenol ethoxylates
		Acetylenic diols
		Alkyl aryl sulfonates
		Sulfosuccinates

## 5. Ageing Behaviour and Material Degradation

Some modern plastic-type materials, including polymer-based paints, are believed to be more prone to rapid degradation relative to some of the traditional materials in collections [54,55]. Poly(vinyl acetate) can degrade in several different ways, including chemical degradation (hydrolysis and oxidation), physical degradation (UV light ageing), and biological degradation (microbial attack) [92,93,94,95,96,97]. However, due to the relative newness of these materials and their complex chemical composition, there is a limited body of knowledge regarding the degradation mechanism of poly(vinyl acetate) paints and the environmental parameters which may influence the degradation processes. In addition, it is also not certain how these different degradation mechanisms, such as hydrolysis and light ageing, may influence each other, and which mechanisms become dominant under which environmental conditions. Consequently, there are some visible signs of degradation in many artworks made of poly(vinyl acetate) paints [55]. These signs of degradation may consist of colour shifts, surface changes caused by plasticiser and surfactant deposits, craquelure, and the release of volatile compounds, such as acetic acid. In the past decade, several researchers have carried out various forms of accelerated and natural ageing to study the different degradation mechanisms of poly(vinyl acetate) paints and resins, with the aim of predicting how these paints change in order to help professionals make informed decisions about optimal storage, display, and treatment conditions.

The three main degradation pathways of poly(vinyl acetate) are presented in more detail in the following subsections.

### 5.1. Hydrolysis

Most modern polymers are susceptible to degradation when exposed to water, as some functional groups in the polymer chain, such as the ester groups in vinyl paints, can be hydrolysed in the presence of water [51]. Consequently, this can cause the breakage of the polymer chain and the release of hydrolysed monomer units as well as small molecules from the side chains. When the poly(vinyl acetate) polymer chain is hydrolysed, it forms poly(vinyl alcohol) (PVOH) and releases acetic acid as a side product [95,96,98]. This hydrolytic reaction was first discovered by Hermann and Haehnel in the 1920s when the acetate groups were hydrolysed by alcohols, such as methanol, in the presence of alkali catalysts. The acetate groups can be hydrolysed to various degrees under alkaline conditions, and newly synthesised poly(vinyl alcohol) is then precipitated, washed, and dried [95,99,100]. In the 1930s, several authors studied poly(vinyl acetate) chemical degradation upon hydrolysis and repeated acetylation. They discovered that this degradation is mostly manifested through the loss of the poly(vinyl acetate) molecular weight and viscosity, due to the presence of impurities or the side chain reactions which created branched acetals [25,96,101].

To date, only a few heritage studies have focused on the effects of moisture on poly(vinyl acetate) paints. Ferreira et al. monitored the formation of poly(vinyl alcohol) during the degradation process using spectroscopic techniques, such as Fourier transform infrared spectroscopy (FTIR), and computational methods in order to predict the development of poly(vinyl alcohol) during acid hydrolysis [102,103]. Furthermore, some authors noted the visible signs of degradation, such as a strong acidic smell from the released acetic acid, of poly(vinyl acetate) emulsion paints that were stored in tightly closed containers, such as paint cans [8,13,37]. They attributed this effect to the acid-catalysed hydrolysis of poly(vinyl acetate) in water, which results in the development of poly(vinyl alcohol). To the best of the authors’ knowledge, the effect of fluctuating relative humidity on poly(vinyl acetate) paint films has not been studied, though they are likely to respond similarly to acrylic emulsion paints, i.e., causing changes in the paint’s mechanical properties such as stiffness, and may also promote the movement of additives within films [104,105,106,107,108,109,110].

### 5.2. Photochemical and Oxidative Degradation

In addition to hydrolytic degradation, poly(vinyl acetate) is sensitive to other environmental parameters, such as excessive levels of light, especially in the ultraviolet region, and/or oxygen. Research on the photodegradation of poly(vinyl acetate) began in the 1970s when David et al. discovered that poly(vinyl acetate) resin becomes partially insoluble in benzene after exposure to UV light [111]. The authors explained this behaviour as being a consequence of the cross-linking and chain scission reactions of the polymer. In addition, they observed that in the presence of oxygen, the rate of the photodegradation of poly(vinyl acetate) was reduced. Several authors also calculated the quantum yield of volatile species formation, e.g., acetic acid, carbon monoxide and dioxide, and methane, after the UV light exposure of poly (vinyl acetate) thin films. The measurements were conducted in both air and a vacuum and at room temperature and higher (45 °C) [112,113]. Buchanan et al. also noted aldehyde formation and changes in the molecular mass distribution between the soluble fraction and the gel formation of photoaged poly(vinyl acetate), indicating that both cross-linking and chain scission influence the poly(vinyl acetate) photodegradation to similar degrees [114]. Consequently, they proposed two poly(vinyl acetate) photodegradation mechanisms, which accounted for the aldehyde formation [115]. Vaidergorin et al. also monitored the UV light degradation of poly(vinyl acetate) films of various molecular weights in a vacuum using infrared and UV spectroscopy [116]. They concluded that the degradation proceeds through side chain acetate group elimination and the formation of polyenes, in addition to cross-linking and main chain scission. For oxidative degradation, the reported studies are much more rare. Madras et al. investigated the oxidative degradation of poly(vinyl acetate) in solution using benzoyl peroxide. A comparison of molecular weights indicated that oxidative degradation occurs primarily through the chain scission mechanism [117].

At present, it is presumed that the photochemical degradation of poly(vinyl acetate) degradation occurs through the Norrish II mechanism [54,118,119,120], where chain scission is the main reaction pathway. As the reaction proceeds, volatile species such as acetic acid, methane, carbon dioxide, and monoxide are formed and released, and double –C=C– bonds in the main polymer chain are formed [16,51,121]. The loss of acid, in addition to the plasticiser loss, leads to physical changes in the poly(vinyl acetate) material, such as changes in colour (yellowing and loss of transparency) and the loss of mechanical properties, which causes the material to become brittle and or powdery [85]. Toja et al. discovered that thermal ageing at 60 °C has a more pronounced effect on polymer degradation, leading to the rapid loss of (external) phthalate plasticisers and deacetylation, which causes the formation of –C=C– bonds in the polymer backbone [54]. For photooxidative (light) ageing, the deacetylation process was not observed, and the plasticiser loss occurred less rapidly, with some residual plasticiser remaining detectable in the polymer after ageing.

Several researchers have observed a different response to ultraviolet (UV) photoageing between different poly(vinyl acetate) products, such as pure poly(vinyl acetate) homopolymers, resins, and a range of paints [9,16,18]. The results suggested that the pure vinyl homopolymers tended to be very resistant to light ageing, with no colour change observed. The poly(vinyl acetate) resins based on copolymers and external plasticisation, however, showed a slight yellowing effect and a loss of plasticiser. Other groups noted that the morphological changes in poly(vinyl acetate) paint films were minimal and that these paints showed noticeable resistance to light ageing [51,53]. Silva et al. concluded that different poly(vinyl acetate) paints and resins react differently to photodegradation [51]. While the Lefranc and Bourgeois artists’ Flashe paints, which contained vinyl versatates as a copolymer, did not show loss of mechanical properties after UV ageing, pure poly(vinyl acetate) resins with the addition of dibutyl phthalate plasticiser, such as Conrayt, or those copolymerised with butyl maleate, such as Mowilith DMC2, showed significant changes in mechanical properties. This resulted in either increased stiffness of the paint films due to plasticiser loss and chain scission for Conrayt or polymer chain cross-linking for Mowilith DMC2.

Several studies focused on finding the dominant degradation pathway that occurs during light degradation; however, they arrived at different conclusions. In two studies conducted by Ferriera et al., it was noted that the dominant degradation pathway under the experimental degradation conditions, i.e., light exposure at λ ≥ 300 nm, was chain scission, without observable side group elimination [13,16]. Another study found that poly(vinyl acetate) paints degraded through both main chain scission and side chain reactions [116,122]. Wei et al. noted an increase in acetic acid and the loss of plasticisers after the photoageing process [119]. Down et al. carried out a comparison of light and dark (thermal) ageing between acrylics and poly(vinyl acetate) adhesives, where the poly(vinyl acetate) was more susceptible to degradation [39]. This group also observed that poly(vinyl acetate) adhesives tended to become more acidic, release larger amounts of acid volatiles, show less flexibility, and yellow more rapidly. In addition, several authors reported that the migration and loss of paint additives, such as poly(ethylene oxide) (PEO) surfactants and phthalate plasticisers, were also observed during UV light ageing [9,53,122,123].

The photodegradation mechanism of pigmented vinyl acetate paints can cause a visible colour change, such as colour fading. Therefore, several studies have focused on the influence of other paint components, such as inorganic and organic pigments, on the photodegradation of poly(vinyl acetate) paints. There are some inconsistencies in the outcomes of these studies, and the clear effect of pigments on the photostability of poly(vinyl acetate) paints has not been fully established. Doménech-Carbó et al. reported a more noticeable polymer degradation for poly(vinyl acetate) paint formulations containing organic pigments, such as Senegal yellow (monoazo pigment yellow 3) [122]. Melchiorre Di Crescenzo et al. found that poly(vinyl acetate) formulations containing ultramarine blue pigment showed earlier signs of photodegradation [120]. The presence of other pigments in poly(vinyl acetate) paints and resins, such as the lithopone and calcium carbonate mixture, burnt umber, cobalt blue, cadmium red dark, and titanium white, were all reported to have a destabilising effect [9,119].

Several other authors reported that there were no observable effects on the photodegradation of poly(vinyl acetate) from the presence of pigments. The influence of metals arising from inorganic pigments on the degradation of poly(vinyl acetate) was studied by Ferreira et al. [16]. The authors noted that the presence of pigments on the photostability of poly(vinyl acetate) does not have a large effect, even when using photocatalytic pigments, such as TiO_2_ and Fe_2_O_3_, in formulations. Another study conducted by Silva et al. came to a similar conclusion, indicating that there was no observable influence of a range of different pigments on the loss of the mechanical properties of poly(vinyl acetate) paints exposed to UV light ageing [51]. However, this effect is not clear, as other researchers noted that the presence of some pigments can have a stabilising effect on the photothermal ageing of poly(vinyl acetate)-based paints [9,53,119]. De Sá et al. reached this conclusion, finding that the presence of TiO_2_ pigment in its rutile form reduces the loss of surfactants from photoaged vinyl films [53]. This is the opposite behaviour to that of acrylic paints, as reported by Naude et al. [124]. Pereira also noted that paint formulations with TiO_2_ were more stable during photoageing [9]. Wei et al. detected that poly(vinyl acetate) mixed with nickel azo yellow pigments showed higher resistance to photodegradation compared to the formulations that contained pigments such as cadmium red, cobalt blue, titanium white, and burnt umber [119]. It is clear that for vinyl emulsion paints, the relationships between the solids content, type, and amounts, as well as between the additive types and amounts, on both paint film quality and ageing behaviour, clearly requires further research, in addition to investigations as to how this may influence the response to cleaning of a given paint film.

### 5.3. Thermal Degradation

In addition to photochemical degradation, poly(vinyl acetate) is known to degrade when exposed to high temperatures [38,125,126,127]. The influence of temperature on the thermal degradation of poly(vinyl acetate) under a vacuum was first reported by Grassie et al. [126,127]. They noted that the initial degradation of the vinyl polymer chain generally starts in the temperature range from 190 to 300 °C and that the mechanisms of thermal degradation are side chain scission and the loss of acetic acid, which causes the formation of double bonds (polyene) in the main polymer chain [128]. Bataille et al. concluded that the release of acetic acid subsidies at temperatures higher than 350 °C, while at higher temperatures the degradation of acetic acid to methane and carbon dioxide occurs [129]. This reaction occurs in the absence of free radicals. At temperatures above 400 °C, the polymer backbone starts to decompose with a subsequent release of various aromatic hydrocarbon species [125]. Servotte et al. noted that the release of acetic acid is followed by the cross-linking of the remaining polymer chains [128]. Later on, Ballistreri et al. reported a two-step thermal degradation mechanism: at temperatures below 310 °C, the mechanism is dominated by the release of acetic acid, while at higher temperatures there is a dominant release of aromatic hydrocarbons due to the polyene degradation [130]. While Grassie and Bataille noted that the rate of thermal degradation depends on the intrinsic properties of poly(vinyl acetate), such as molecular weight, Servotte et al. did not observe any difference in the degradation rates between the poly(vinyl acetate) resins of different molecular weights. There are several theories as to how the thermal degradation reaction is catalysed after the release of acetic acid. Several authors have stated that the newly formed double bonds have an autocatalytic effect on poly(vinyl acetate) degradation, which initiates the further release of acetic acid and the formation of double bonds [125,126]. However, some pose that the presence of released acetic acid has an autocatalytic effect on further poly(vinyl acetate) degradation [131,132,133]. Although the effect of temperature fluctuation on vinyl paints is not widely explored, temperature fluctuations affect vinyl paint mechanical properties as expected: above *T_g_*, paint films become soft and can attract dirt and dust, while below *T_g_* paint films become brittle and susceptible to cracking [9].

## 6. Analytical Methods for the Characterisation of Poly(Vinyl Acetate) Materials

Several groups have focused on the analysis of poly(vinyl acetate) products and their properties, including pure resins and paints [18,58,61,84,85,91,123,134,135,136]. Most of these studies included a multi-analytical approach to obtain comprehensive information on paint and resin formulations. As noted, modern paints, including poly(vinyl acetate), are known for their complex chemical compositions, which are often proprietary in nature, and many of the constituents are present in low amounts, which can prove analytically challenging. Many techniques have been employed to detect the chemical composition of modern poly(vinyl acetate)-based paints; however, the two most widely used are Fourier transform infrared (FTIR) spectroscopy and pyrolysis–gas chromatography–mass spectrometry (PyGCMS) [5,137]. These two techniques are considered standard for analysing polymeric materials and have been widely employed for the analysis of plastics, various modern paints, and oil paints. These methods provide information on polymer composition and additives and facilitate the monitoring of degradation products and the exploration of the effects of conservation treatments through the analysis of paint extracts and other fractions.

FTIR is generally suitable and is used for detecting primary polymer composition as well as the presence of inorganic and organic pigments, extenders, and additives (e.g., waxes and plasticisers) [5,138]. In the past, FTIR has been employed in many different modes, such as attenuated total reflectance (ATR) [9,18,22,34,51,54,91,119,120,121,122,123,134,139], micro-FTIR [8,9,16,17,18,31,32,62,102,123,140], thermal analysis FTIR [125], and the external reflectance mode [10,18], to analyse artistic materials and/or works of art made of vinyl paints.

PyGCMS facilitates the separation and detection of various polymeric components, as well as some pigments and a range of additives, all of which are generated through the thermal degradation of the sample, and it is the global method of choice for studying different additives in paints and resin formulations. Poly(vinyl acetate) can be differentiated from other polymers by the presence of two characteristic PyGCMS peaks: acetic acid (*m/z* = 43, 45, and 60) and benzene (*m/z* = 78) [5,141]. The presence of both peaks needs to be established in order to be certain that the analysed material is poly(vinyl acetate), as acetic acid is also a pyrolytic product of other polymers, such as cellulose acetate [5]. These two compounds are created through side chain scission and polymer backbone rearrangement during thermal degradation. The acetic acid and benzene peaks are easily differentiated; while acetic acid shows a strong fronting peak, the benzene peak elutes slightly later and appears sharper. In addition to the key polymer degradation markers, the presence of some additives, such as plasticisers (both internal and external), surfactants, and organic colourants, can be detected via PyGCMS. Using this method, the external plasticisers presented in Table 1, such as dibutyl phthalate (DBP), diethyl phthalate (DEP), diisobutyl phthalate (DIBP), bis(2-ethylhexyl) phthalate (DEHP), dipropylene glycol dibenzoate (DPGDB), diethylene glycol dibenzoate (DEGDB), and triphenyl phosphate (TPP), and internal plasticisers, such as vinyl versatates (VeoVa), have been detected in poly(vinyl acetate) paints and resins [18,58,61,61,84,85,91,135].

Raman spectroscopy has been an established method to analyse the organic and inorganic pigments and extenders present in modern paints [8,10,11,142,143,144,145]. The binding media analysis and identification by Raman is, however, often not easily possible due to the high fluorescence effect [10,146]. However, several Raman techniques, such as surface-enhanced Raman spectroscopy and surface-enhanced resonance Raman spectroscopy, have been employed in the last decade to analyse highly fluorescent paint materials [146]. Furthermore, several authors used Raman spectroscopy to monitor the light degradation of poly(vinyl acetate) resins and also to detect plasticizers in poly(vinyl acetate) resins [18,91].

Several researchers have used other standard polymer investigative techniques to characterise poly(vinyl acetate) paints, to analyse degradation products, and to assess the effects of surface treatments on paint films. These include the use of size exclusion chromatography [16,18] and gel permeation chromatography [34] to study changes in the molecular weight of vinyl polymers during photochemical ageing; mechanical testing [22,34,51,61,62,122] to explore properties such as paint stiffness/flexibility; thermal analysis (thermogravimetric analysis, differential scanning calorimetry, and dynamic mechanical analysis) [54,125] to explore physical properties such as glass transition temperature; dynamic light scattering to determine vinyl particle size [13,16]; atomic force microscopy [9,22,53,122,140] and scanning electron microscopy for surface morphology and scanning electron microscopy with energy-dispersive X-ray spectroscopy for pigment identification; and UV–Vis spectroscopy [16,122], fluorescence spectroscopy [54], laser-induced fluorescence (LIF) [11], and nuclear magnetic resonance [54,147,148] for the chemical characterisation of vinyl polymer, pigments, and/or plasticisers. The key techniques used in these studies are presented in more detail in Table 2.

## 7. Conservation Issues and Cleaning Effects

Over the last two decades, the effects of different cleaning treatments on the properties of modern paints have been a key area of heritage scientific research. There remain, however, a variety of challenges encountered in the conservation of modern painted works of art, and the risks associated with cleaning procedures can still prohibit treatment [5,90,104,140,151,152,153,154]. These painted works are often unvarnished and intentionally unprotected, and they can be made with several types of paint/material, all of which can include complex chemical compositions and represent a range of possible degradation pathways. In particular, the sensitivity of waterborne modern paints to cleaning solvents, including water, and to excessive soiling and dust accumulation, due to their inherently soft nature (low *T_g_*), as well as the associated unwanted changes in gloss, texture, and surface quality during the cleaning process, represent particular challenges in the cleaning of modern and contemporary works of art [90,153,155,156,157].

The current cleaning strategies for poly(vinyl acetate) paints are generally comparable to those used on modern oils and acrylic emulsions. This includes a range of dry methods, as well as ‘simple’ aqueous systems (adjusted and buffered waters, surfactants, chelating agents) applied with cotton swabs or sponges, as well as some low-swelling organic solvents, such as hydrocarbons, alcohols, and silicones [158,159,160]. In the last decade or so, there has been an increase in the use of microemulsions and gel systems for the cleaning of solvent-sensitive surfaces, including modern paints [161,162,163]. The assessment of the cleaning results can include visual observation, microscopic and surface observations using SEM and AFM, colour and gloss measurements, mechanical testing, chemical characterisation of the surfaces before and after cleaning (FTIR and PyGCMS), water absorption tests, and the use of star diagrams to capture valuable empirical and haptic observations [153,164,165].

Compared to acrylic emulsion paints, however, the research into the effects of solvents on poly(vinyl acetate) paints remains relatively lean. To date, the studies have focused on exploring the effects of different solvent systems, including immersion testing and swab rolling [9,18,22,51,62]. Zumbühl et al. evaluated 50 different solvents and their effects on poly(vinyl acetate)-VeoVa paints, using immersion methods for 1 and 15 min [62]. The effect of the solvents on the film surface morphology was observed using scanning electron microscopy, and the paint extracts were chemically characterised using FTIR spectroscopy. The results indicated that highly polar solvents, such as water, methanol, and formamide can cause the swelling of poly(vinyl acetate) paints copolymerised with VeoVa; this is due primarily to the presence of soluble polar additives. Except for methanol, most other alcohol solutions did not show a swelling effect on the vinyl paints assessed [62]. In addition, the immersion process for polar solvents, such as water and formamide, resulted in the extraction of some additives, such as the poly(ethylene oxide)-based surfactants, as has been repeatedly shown for acrylic emulsion paints [104,151,166]. The solvent immersions also caused the films to become slightly more elastic compared to the untreated poly(vinyl acetate)-VeoVa paint film, which showed poor mechanical properties due to the high amounts of additives, which inhibited the film formation. The most probable explanation for this increase in the elasticity of paint films is the plasticising effect of some solvent systems, such as the water-based ones [151]. However, some authors reported different observations for this type of poly(vinyl acetate) paint. Silva et al. reported that the lower detected amounts of PEO-type surfactants in poly(vinyl acetate)-VeoVa paint formulations correlated with the higher flexibility of paint films and vice versa [61]. Doménech-Carbó et al. explained this inherent stiffness of the commercial poly(vinyl acetate)-VeoVa paints due to their higher pigment content [122]. It is clear that the effect of solvent treatments on poly(vinyl acetate) paints and their additives and the subsequent changes in the paint’s mechanical properties is not entirely clear, and this needs more research. In addition, it is generally accepted that paints which have been extracted using solvent systems tend to be stiffer, as established for oil and acrylic paints; therefore, the conclusions from these studies are not definitive [151]. Except for water and formamide, the immersion in other solvents led to a further decline in the mechanical properties of poly(vinyl acetate)-VeoVa paints, as measured through tensile testing, when compared to untreated poly(vinyl acetate) paints [30,62]. In comparison, non-polar and hydrocarbon solvents, such as mineral spirits and ligroin, did not cause swelling; however, the longer evaporation times of these solvents caused a plasticising effect on the paint film [22,62].

As is the case for acrylic emulsion paints, poly(vinyl acetate) films remain sensitive to aqueous and polar solvents due to the nature of the film formation process, the inherent softness of wetted paint films, and the presence of additives, many of which, such as plasticisers and surfactants, have some solvent solubility. The solubility of some common additives found in vinyl products is presented in Table 3, which suggests that although phthalate plasticisers have low solubility in water, PEO-surfactants are, as expected, much more vulnerable to extraction with water. In addition, both plasticisers and surfactants show solubility in numerous organic solvents. Several authors have reported the loss of various additives, such as polyethylene oxide (surfactants), poly(vinyl alcohol) (protective colloid), and cellulose ether (thickener), as well as vinyl chains, during immersion studies (5, 10, 20 min, and 12 h) using water and organic solvents [9,22]. The presence of extracted additives was observed using FTIR and PyGCMS methods [9,22]. A subsequent decline in the mechanical properties after immersion testing was also noted, demonstrating a potential plasticising effect of these additives (in addition to the water itself) on the poly(vinyl acetate) paint films [9,22,58,107,167]. However, this could also be caused by the leaching of polymer chains; visible changes in mechanical and surface properties were observed in the samples immersed in solvents such as acetone and ethanol—and caused the extraction of polymer chains and phthalate plasticisers [9,22]. Pereira et al. also noted that light-aged poly(vinyl acetate) white (lithopone) paint was more resistant to water immersion than unaged poly(vinyl acetate) white paint [9].

To date, a limited number of studies have focused on the use of gels and microemulsions in the cleaning of poly(vinyl acetate) paints, including Vanzan (xanthan gum), Klucel G (modified cellulose), Gellan, Agarose, Agar-agar, and Nanorestore Peggy gels, and an emulsion mixture of ligroin in water with nonionic polyoxyethylene surfactant [12,18,22,51]. In addition to the assessment of the conservation treatment risk and effects, the long-term stability of poly(vinyl acetate) paints is also widely unknown, with only a few general statements available, discussing how vinyl paints are more prone to weathering than acrylic paints [5,18]. Thus far, the effects of environmental conditions on the degradation pathways of poly(vinyl acetate) resins and paints have focused mostly on light (visible and UV) ageing, while the influence of relative humidity and temperature fluctuations (and combinations of these), as well as the effects of pollutants, on film stability has not been the subject of extensive study.

## 8. Conclusions

The development of modern synthetic polymer paints and their use by artists has led to increased interest in the degradation and conservation of works of art containing these paints. However, for poly(vinyl acetate) paints, the knowledge of paint properties and the tailoring of conservation strategies remain relatively limited when compared to acrylic emulsion and modern oil paints (though these paints also require further research).

Poly(vinyl acetate) as a resin and unpigmented emulsion is vulnerable to light, thermal degradation, and hydrolysis, and poly(vinyl acetate) paints are also highly sensitive to changes in colour and gloss when exposed to light and are vulnerable to a range of solvents. Most of the published scientific research has focused on polymer characterisation, light ageing, and the effect of pigments on the ageing process. Thermal and hydrolytic ageing and microbial attack have been the focus of only a handful of studies, and the assessment of cleaning effects and the development of low-risk strategies for the cleaning of poly(vinyl acetate) painted works of art remain a priority. Poly(vinyl acetate) paints are vulnerable to polar solvent systems, including water; this is partly due to the presence of hydrophilic additives in paint formulations. Non-polar solvents tend to cause lower swelling; however, some of the paint additives may also be vulnerable to extraction with these solvents. The use of gels and other ways of confining solvent volume and diffusion into paint films is likely to prove beneficial for the cleaning of poly(vinyl acetate) paints, as has been the case for acrylics, oils, and other modern paints.

In contemporary conservation, contributing to the development of low-risk conservation and preservation strategies necessarily involves the characterisation of paint components, contextual and historic studies, and the assessment of cleaning systems and ageing effects, as well as the development, modification, and assessment of cleaning systems and their use on case study works of art. It is hoped, therefore, that this review provides a helpful assessment of the current knowledge of poly(vinyl acetate) paints and forms a basis for new research and innovations in conservation practice.

## Figures and Tables

**Table 2:** Summary of analytical techniques employed for the investigation of poly(vinyl acetate) materials.

Material Aspect Investigated	Techniques Employed	Research Aims	References
Polymer, colourants, and additives identification	Fourier transform infrared spectroscopy (FTIR)	Chemical characterisation of the vinyl binder, colourants, and additives Monitor photooxidative and thermal degradation of vinyl polymer Monitor polymer or additive extraction during cleaning treatments	Ferreira et al. [8,16,32,102] Pereira et al. [9] Mancini et al. [10] Carter et al. [17,31] Viana [18] Doménech-Carbó et al. [22,122] Alderson et al. [34,139] Silva et al. [51] Toja et al. [54,123] Zümbuhl et al. [62] De Sá et al. [91] Wei et al. [119] Melchiorre Di Crescenzo et al. [120] Pintus et al. [121] Holland et al. [125] Izzo et al. [134] Ormsby et al. [140]
	Pyrolysis–gas chromatography–mass spectrometry (PyGCMS)	Chemical characterisation of vinyl binder, colourants, and additives (plasticisers, surfactants)	Ferreira et al. [8] Pereira et al. [9] Carter et al. [17,31] Viana [18] Doménech-Carbó et al. [22] Silva et al. [51,58,61,85] Doménech-Carbó et al. [84,97,122] Wei et al. [119] Pintus et al. [121,135] Toja et al. [123] Izzo et al. [134] Schossler et al. [136] Ormsby et al. [140] Learner [149] Peris-Vicente et al. [150]
	Nuclear magnetic resonance (NMR)	Monitor structural changes in polymer backbone during thermal and photooxidative ageing	Toja et al. [54]
		Chemical characterisation of the poly(vinyl acetate) restoration layer	Kehlet et al. [147]
		Structural characterisation of commercial poly(vinyl acetate) products	De Souza and Tavares [148]
	Raman spectroscopy	Chemical characterisation of poly(vinyl acetate) paints (pigments, extenders)	Ferreira et al. [8]
		Chemical characterisation of poly(vinyl acetate) paintings (organic and inorganic pigments)	Mancini et al. [10]
		Chemical characterisation of poly(vinyl acetate) paintings (organic and inorganic pigments)	Spizzichino et al. [11]
		Chemical characterisation of poly(vinyl acetate) resins after light ageing	Viana [18]
		Chemical characterisation of poly(vinyl acetate) homopolymer, and plasticised and non-plasticised resins (polymer and plasticisers)	De Sá et al. [91]
	Ultraviolet–visible (UV–Vis) spectroscopy	The reflectance spectrum of the light-aged poly(vinyl acetate) paint films	Ferreira et al. [16]
		The reflectance spectrum of the light-aged poly(vinyl acetate) paint films	Doménech-Carbó et al. [122]
	Fluorescence spectroscopy	Fluorescence emission of poly(vinyl acetate) films after thermal-oxidative and photooxidative ageing	Toja et al. [54]
	Laser-induced fluorescence (LIF)	Chemical characterisation of poly(vinyl acetate) paintings (vinyl binder and pigments)	Spizzichino et al. [11]
	Scanning electron microscopy energy dispersive X-ray spectroscopy (SEM-EDX)	Elemental analysis of the poly(vinyl acetate) paint films	Melchiorre Di Crescenzo et al. [120]
		Elemental analysis of the poly(vinyl acetate) paint films	Doménech-Carbó et al. [122]
Physical properties	Mechanical (tensile) testing	The effect of different cleaning strategies (immersion, swabbing, gels, and microemulsions) on the tensile properties of the paint films	Doménech-Carbó et al. [22]
		Mechanical testing of the poly(vinyl acetate) paint films after light ageing (λ = 340 nm)	Alderson et al. [34]
		Mechanical testing of the daylight- and UV light-aged poly(vinyl acetate) paint films, and the effect of cleaning	Silva [51]
		Mechanical testing of the poly(vinyl acetate) paint films and its dependence on the paints’ additives	Silva et al. [61]
		Mechanical testing of the poly(vinyl acetate) paint films and the effect of cleaning treatments	Zumbühl et al. [62]
		Mechanical testing of the daylight- and UV light-aged poly(vinyl acetate) paint films	Doménech-Carbó et al. [122]
	Thermal analysis (thermogravimetric analysis, differential scanning calorimetry, or dynamic mechanical analysis)	Monitor glass transition temperature during photooxidative (λ ≥ 290 nm) and thermal-oxidative ageing (60 ± 2 °C) of plasticised and unplasticized poly(vinyl acetate) films	Toja et al. [54]
		Monitor mass loss during thermal degradation of poly(vinyl acetate) films	Holland and Hay [125]
	Dynamic light scattering (DLS)	Determination of particle size and its influence on the cross-linking of polymer chains	Ferreira et al. [13,16]
	Size exclusion chromatography (SEC)	Determination of M_w_ of poly(vinyl acetate) paints and its change during ageing	Ferreira et al. [13,16]
		Determination of M_w_ of poly(vinyl acetate) resins and its change during ageing	Viana et al. [18]
	Gel permeation chromatography	Determination of M_w_ of poly(vinyl acetate) resins	Alderson et al. [34]
Surface analysis	Atomic force microscopy (AFM)	Surface analysis of poly(vinyl acetate) resins and paints after light ageing and cleaning treatments	Pereira [9]
		Surface analysis of poly(vinyl acetate) paints before and after cleaning treatments	Doménech-Carbó et al. [22]
		Surface changes of pure poly(vinyl acetate), resin, and white paint after light ageing (λ ≥ 300 nm)	De Sá et al. [53]
		Surface changes of the poly(vinyl acetate) paint films after daylight and UV light ageing	Doménech-Carbó et al. [122]
	Scanning electron microscopy (SEM)	Surface morphology of the poly(vinyl acetate) paint films exposed to daylight and UV light ageing and after cleaning treatments	Doménech-Carbó et al. [22,122]
		Surface morphology of the poly(vinyl acetate) paint films during immersion and solvent cleaning	Silva [51]
		Surface morphology of the poly(vinyl acetate) paint films during immersion and solvent cleaning	Zumbühl et al. [62]
		Surface morphology of poly(vinyl acetate) paint films after light ageing (295 < λ < 370 nm)	Melchiorre Di Crescenzo et al. [120]

**Table 3:** Solubility properties of the common additives found in poly(vinyl acetate) products.

Additives	Solubility in Water at 25 °C (g/L)	Solubility in Other Solvents
Dibutyl phthalate 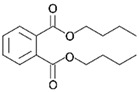	0.01 [168,169]	Soluble in alcohol, benzene, and ether [168]
Diethyl phthalate 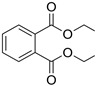	1 [170]	Soluble in alcohol, acetone, benzene, ketones, ethers, esters, aliphatic solvents, and aromatic hydrocarbons [170]
Bis(2-ethylhexyl) phthalate 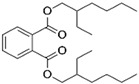	0.0003–0.0004 [171]	Miscible with most common organic solvents [171]
Diisobutyl phthalate 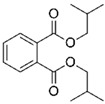	0.02 [169,172]	Soluble in ethanol, ether, and benzene [173]
Triphenyl phosphate 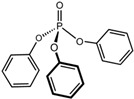	0.0019 [174]	Soluble in acetone, benzene, chloroform, and ether; moderately soluble in ethanol [174]
Poly(ethylene oxide) 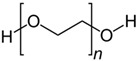	~550 (depends on polymer M_w_) [175]	Soluble in aliphatic ketones, alcohols, chloroform, glycol ethers, esters, and aromatic hydrocarbons; insoluble in ether and most aliphatic hydrocarbons [175]

## Data Availability

Not applicable.

## Abbreviations

AFM	atomic force microscopy
DBP	dibutyl phthalate
DEP	diethyl phthalate
DEHP	bis(2-ethylhexyl) phthalate
DIBP	diisobutyl phthalate
DLS	dynamic light scattering
FTIR	Fourier transform infrared spectroscopy
LIF	laser-induced fluorescence
M_w_	molecular weight
NMR	nuclear magnetic resonance
PEO	poly(ethene oxide)
PVAc	poly(vinyl acetate)
PVOH	poly(vinyl alcohol)
PyGCMS	pyrolysis–gas chromatography–mass spectrometry
SEC	size exclusion chromatography
SEM	scanning electron microscopy
SEM-EDX	scanning electron microscopy energy dispersive X-ray spectroscopy
T_g_	glass transition temperature
TPP	triphenyl phosphate
UV	ultraviolet
UV–Vis	Ultraviolet–visible spectroscopy
VeoVa	vinyl versatate esters

## References

[1] Crook J., Learner T. (2000). The Impact of Modern Paints.

[2] Learner T. (2007). Modern paints: Uncovering the choices. Modern Paints Uncovered: Proceedings from the Modern Paints Uncovered Symposium.

[3] Learner T. (2000). A review of synthetic binding media in twentieth-century paints. Conservator.

[4] Izzo F.C., van den Berg K.J., van Keulen H., Ferriani B., Zendri E., van den Berg K.J., Burnstock A., de Keijzer M., Krueger J., Learner T., de Tagle A., Heydenreich G. (2014). Modern Oil Paints—Formulations, Organic Additives and Degradation: Some Case Studies. Issues in Contemporary Oil Paint.

[5] Learner T. (2004). Analysis of Modern Paints.

[6] Burnstock A., van den Berg K.J., van den Berg K.J., Burnstock A., de Keijzer M., Krueger J., Learner T., de Tagle A., Heydenreich G. (2014). Twentieth Century Oil Paint. The Interface Between Science and Conservation and the Challenges for Modern Oil Paint Research. Issues in Contemporary Oil Paint.

[7] Hillary S., Campbell K., Carlisle M., Khanjian H., Learner T., Schilling M. (2013). The early use of synthetic emulsion paints by New Zealand artists. AICCM Bull..

[8] Ferreira J.L., Ávila M.J., Melo M.J., Ramos A.M. (2013). Early aqueous dispersion paints: Portuguese artists’ use of polyvinyl acetate, 1960s–1990s. Stud. Conserv..

[9] Pereira A.I.M.L. (2015). The Perfect Paint in Modern Art Conservation: A Comparative Study of 21st Century Vinyl Emulsions. Ph.D. Thesis.

[10] Mancini D., Percot A., Bellot-Gurlet L., Colomban P., Carnazza P. (2021). On-site contactless surface analysis of modern paintings from Galleria Nazionale (Rome) by reflectance FTIR and Raman spectroscopies. Talanta.

[11] Spizzichino V., Angelini F., Caneve L., Colao F., Corrias R., Ruggiero L. (2015). In situ study of modern synthetic materials and pigments in contemporary paintings by laser-induced fluorescence scanning. Stud. Conserv..

[12] Schmid A. Cleaning of Matte White Polyvinyl Acetate Paint with Nanorestore Gels®. Proceedings of the AIC/SPNHC Joint Virtual Annual Meeting.

[13] Ferreira J.L.A. (2011). Liaisons Dangereuses, Conservation of Modern and Contemporary art: A Study of the Synthetic Binding Media in Portugal. Ph.D. Thesis.

[14] Croll S. (2007). Overview of Developments in the Paint Industry since 1930. Modern Paints Uncovered: Proceedings from the Modern Paints Uncovered Symposium.

[15] Dredge P., Carter A., Osmond G. (2020). Sidney Nolan: The Artist’s Materials.

[16] Ferreira J.L., Melo M.J., Ramos A.M. (2010). Poly(vinyl acetate) paints in works of art: A photochemical approach: Part I. Polym. Degrad. Stab..

[17] Carter A., Osmond G., Ormsby B. (2014). Ian Fairweather and water-based emulsion house paints in Australia 1950–64. AICCM Bull..

[18] Viana C.D.R. (2022). Are All Vinyl Paints the Same?. Masters Thesis.

[19] (1977). ‘Peter Grimes’s Apprentice‘, Sir Sidney Nolan. https://www.tate.org.uk/art/artworks/nolan-peter-grimess-apprentice-t03560.

[20] Stols-Witlox M., Ormsby B., Gottsegen M. (2012). Grounds, 1400–1900. The Conservation of Easel Paintings.

[21] Riley B., Moorhouse P. (2003). Bridget Riley.

[22] Doménech-Carboó M.T., Silva M.F., Aura-Castro E., Doménech-Carboó A., Fuster-López L., Gimeno-Adelantado J.V., Kröner S.U., Martínez-Bazán M.L., Más-Barberá X., Mecklenburg M.F. (2010). Multitechnique Approach to Evaluate Cleaning Treatments for Acrylic and Polyvinyl Acetate Paints. Proceedings of the Cleaning 2010 Congress. New Insights into the Cleaning of Paintings.

[23] Gettens R.J., Stout G.L. (1966). Painting Materials: A Short Encyclopaedia.

[24] Klatte F. (1912). Verfahren zur Herstellung Technisch Wertvoller Produkte aus Organischen Vinylestern.

[25] Blaikie K.G., Crozier R.N. (1936). Polymerization of Vinyl Acetate. Ind. Eng. Chem..

[26] Cuthbertson A.C., Gee G., Rideal E.K. (1939). On the polymerization of vinyl acetate. Proc. R. Soc. Lond. A.

[27] Berber H., Ylmaz F. (2013). Emulsion Polymerization: Effects of Polymerization Variables on the Properties of Vinyl Acetate Based Emulsion Polymers. Polymer Science.

[28] Gettens R.J. (1935). Polymerized Vinyl Acetate and Related Compounds in the Restoration of Objects of Art. Tech. Stud. Field Fine Arts.

[29] The History of Flashe. https://www.lefrancbourgeois.com/row/the-history-of-flashe/.

[30] Standeven H.A.L. (2011). Emulsion paints based on synthetic resins. House Paints, 1900–1960—History and Use.

[31] Carter A., Osmond G., Ormsby B. (2014). Characterisation of three early Australian emulsion house paints using FTIR and py-GC/MS. ICOM-CC 17th Triennial Conference: Modern Materials and Contemporary Art.

[32] Ferreira J.L., Melo M.J., Ramos A.M., Avila M.J. (2007). “Eternity is in love with the productions of time”: Joaquim Rodrigo’s classical palette in a vinyl synthetic medium. Modern Paints Uncovered: Proceedings from the Modern apints Uncovered Symposium.

[33] Golden Artist Colors, Inc. PVA Conservation Paints. https://goldenpaints.com/products/custom-products/pva-conservation-paints.

[34] Alderson S., Baade B., Deghetaldi K. (2017). PVA Retouching Colors: A Brief History and Introduction to Golden’s Newly Formulated PVA Conservation Colors. Postprints RECH 4.

[35] Slinckx M., Scholten H.P.H. (1994). Veova-9/(meth)acrylates, a New Class of Emulsion Copolymers. Surf. Coat. Int..

[36] Learner T. (1997). The Characterisation of Acrylic Painting Materials and Implications for Their Use, Conservation and Stability. Ph.D. Thesis.

[37] Horie C.V. (2010). Materials for Conservation: Organic Consolidants, Adhesives and Coatings.

[38] Chelazzi D., Chevalier A., Pizzorusso G., Giorgi R., Menu M., Baglioni P. (2014). Characterization and degradation of poly(vinyl acetate)-based adhesives for canvas paintings. Polym. Degrad. Stab..

[39] Down J.L., MacDonald M.A., Tetreault J., Williams R.S. (1996). Adhesive Testing at the Canadian Conservation Institute: An Evaluation of Selected Poly(Vinyl Acetate) and Acrylic Adhesives. Stud. Conserv..

[40] Firmery G. (2014). Les dispersions de PVAC pour le collage des panneaux peints fragilisés: Réversibilité du collage de joints endommagés. CeROArt.

[41] Cove S., Ellison R., Smithen P., Turnbull R. (2010). Retouching with a PVA Resin Medium. Mixing and Matching—Aproaches to Retouching Paintings.

[42] Kaboorani A., Riedl B. (2011). Improving performance of polyvinyl acetate (PVA) as a binder for wood by combination with melamine based adhesives. Int. J. Adhes. Adhes..

[43] Kaboorani A., Riedl B. (2015). Mechanical performance of polyvinyl acetate (PVA)-based biocomposites. Biocomposites.

[44] Zgueb R., Brichni A., Yacoubi N. (2018). Improvement of the thermal properties of Sorel cements by polyvinyl acetate: Consequences on physical and mechanical properties. Energy Build..

[45] Voskanyan P.S. (2008). Use of Polyvinyl Acetate Plastics in Medicine. Int. Polym. Sci. Technol..

[46] Angelova L.V., Terech P., Natali I., Dei L., Carretti E., Weiss R.G. (2011). Cosolvent Gel-like Materials from Partially Hydrolyzed Poly(vinyl acetate)s and Borax. Langmuir.

[47] Natali I., Carretti E., Angelova L., Baglioni P., Weiss R., Dei L. (2011). Structural and Mechanical Properties of “Peelable” Organoaqueous Dispersions with Partially Hydrolyzed Poly(vinyl acetate)-Borate Networks: Applications to Cleaning Painted Surfaces. Langmuir ACS J. Surf. Colloids.

[48] Angelova L.V., Berrie B.H., de Ghetaldi K., Kerr A., Weiss R.G. (2014). Partially hydrolyzed poly(vinyl acetate)-borax-based gel-like materials for conservation of art: Characterization and applications. Stud. Conserv..

[49] Al-Emam E., Soenen H., Caen J., Janssens K. (2020). Characterization of polyvinyl alcohol-borax/agarose (PVA-B/AG) double network hydrogel utilized for the cleaning of works of art. Herit. Sci..

[50] Ricciotti L., Occhicone A., Manzi S., Saccani A., Ferone C., Tarallo O., Roviello G. (2022). Sustainable Materials Based on Geopolymer–Polyvinyl Acetate Composites for Art and Design Applications. Polymers.

[51] Silva M.F. (2011). Analytical Study of Accelerated Light Ageing and Cleaning Effects on Acrylic and PVAc Dispersion Paints Used in Modern and Contemporary Art. Ph.D. Thesis.

[52] Singh R.P., Heldman D.R., Singh R.P., Heldman D.R. (2014). Chapter 15—Packaging Concepts. Introduction to Food Engineering.

[53] De Sá M.H., Eaton P., Ferreira J.L., Melo M.J., Ramos A.M. (2011). Ageing of vinyl emulsion paints-an atomic force microscopy study. Surf. Interface Anal..

[54] Toja F., Saviello D., Nevin A., Comelli D., Lazzari M., Levi M., Toniolo L. (2012). The degradation of poly(vinyl acetate) as a material for design objects: A multi-analytical study of the effect of dibutyl phthalate plasticizer. Part 1. Polym. Degrad. Stab..

[55] Shashoua Y. (2012). Conservation of Plastics.

[56] King R., Grau-Bové J., Curran K. (2020). Plasticiser loss in heritage collections: Its prevalence, cause, effect, and methods for analysis. Herit. Sci..

[57] Chiantore O., Scalarone D. (2007). The Macro- and Microassessment of Physical and Aging Properties in Modern Paints. Modern Paints Uncovered: Proceedings from the Modern Paints Uncovered Symposium.

[58] Silva M.F., Doménech-Carbó M.T., Osete-Cortina L. (2015). Characterization of additives of PVAc and acrylic waterborne dispersions and paints by analytical pyrolysis–GC–MS and pyrolysis–silylation–GC–MS. J. Anal. Appl. Pyrolysis.

[59] Hayes J., Golden M., Smith G.D. (2007). From Formulation to Finished Product: Causes and Potential Cures for Conservation Concerns in Acrylic Emulsion Paints. Modern Paints Uncovered: Proceedings from the Modern Paints Uncovered Symposium.

[60] Husbands M.J., Standen C.J., Hayward G. (1987). A Manual for Resins for Surface Coatings. 3.

[61] Silva M.F., Doménech-Carbó M.T., Fuster-López L., Mecklenburg M.F., Martin-Rey S. (2010). Identification of additives in poly(vinylacetate) artist’s paints using PY-GC-MS. Anal. Bioanal. Chem..

[62] Zumbühl S., Attanasio F., Scherrer N.C., Muller W., Fenners N., Caseri W. (2006). Solvent action on dispersion paint systems and the influence on the morphology–changes and destruction of the latex microstructure. Modern Paints Uncovered: Proceedings from the Modern Paints Uncovered Symposium.

[63] Brown G.L. (1956). Formation of films from polymer dispersions. J. Polym. Sci..

[64] Felton L.A. (2013). Mechanisms of polymeric film formation. Int. J. Pharm..

[65] GAC Inc. Technical Notes on Drying. Just Paint. https://justpaint.org/technical-notes-on-drying/.

[66] Townsend M. Investigating the Drying Process of Acrylic Color and Gel Medium. Just Paint. https://justpaint.org/investigating-the-drying-process-of-acrylic-color-and-gel-medium/.

[67] Tumosa C.S., Mecklenburg M.F. (2003). Weight Changes in Acrylic Emulsion Paints and the Implications for Accelerated Ageing. WAAC Newsl..

[68] Etemad S.G., Etesami N., Bagheri R., Thibault J. (2002). Drying of latex films of poly(vinylacetate). Dry. Technol..

[69] Keddie J.L., Routh A.F. (2010). Drying of latex films. Fundamentals of Latex Film Formation: Processes and Properties.

[70] Kiil S. (2006). Drying of latex films and coatings: Reconsidering the fundamental mechanisms. Prog. Org. Coat..

[71] Schmidt M., Krieger S., Johannsmann D., Palberg T., Ballauff M. (1997). Film formation of latex dispersions observed with evanescent dynamic light scattering. Optical Methods and Physics of Colloidal Dispersions.

[72] Makan A.C., Spallek M.J., du Toit M., Klein T., Pasch H. (2016). Advanced analysis of polymer emulsions: Particle size and particle size distribution by field-flow fractionation and dynamic light scattering. J. Chromatogr. A.

[73] Kato H., Nakamura A., Takahashi K., Kinugasa S. (2012). Accurate Size and Size-Distribution Determination of Polystyrene Latex Nanoparticles in Aqueous Medium Using Dynamic Light Scattering and Asymmetrical Flow Field Flow Fractionation with Multi-Angle Light Scattering. Nanomaterials.

[74] Carro S., Herrera-Ordonez J., Castillo-Tejas J. (2010). On the evolution of the rate of polymerization, number and size distribution of particles in styrene emulsion polymerization above CMC. J. Polym. Sci. Part A Polym. Chem..

[75] Holthoff H., Borkovec M., Schurtenberger P. (1997). Determination of light-scattering form factors of latex particle dimers with simultaneous static and dynamic light scattering in an aggregating suspension. Phys. Rev. E.

[76] Feng H., Verstappen N.A.L., Kuehne A.J.C., Sprakel J. (2013). Well-defined temperature-sensitive surfactants for controlled emulsion coalescence. Polym. Chem..

[77] Stetefeld J., McKenna S.A., Patel T.R. (2016). Dynamic light scattering: A practical guide and applications in biomedical sciences. Biophys. Rev..

[78] de Bruyn H. (1999). The Emulsion Polymerization of Vinyl Acetate. Ph.D. Thesis.

[79] Lovell P.A., Schork F.J. (2020). Fundamentals of Emulsion Polymerization. Biomacromolecules.

[80] Mills J.S., White R. (2015). The Organic Chemistry of Museum Objects.

[81] Gentekos D.T., Sifri R.J., Fors B.P. (2019). Controlling polymer properties through the shape of the molecular-weight distribution. Nat. Rev. Mater..

[82] Browne E., Worku Z.A., Healy A.M. (2020). Physicochemical Properties of Poly-vinyl Polymers and Their Influence on Ketoprofen Amorphous Solid Dispersion Performance: A Polymer Selection Case Study. Pharmaceutics.

[83] Crescenzo M.M.D., Zendri E., Rosi F., Miliani C. (2013). A Preliminary FTIR-based Exploration of the Surfactant Phase Separation Process in Contemporary Mural Paintings. e-Preserv. Sci..

[84] Doménech-Carbó M.T., Bitossi G., Osete-Cortina L., De La Cruz-Cañizares J., Yusá-Marco D.J. (2008). Characterization of polyvinyl resins used as binding media in paintings by pyrolysis–silylation–gas chromatography–mass spectrometry. Anal. Bioanal. Chem..

[85] Silva M.F., Doménech-Carbó M.T., Fuster-Lopéz L., Martín-Rey S., Mecklenburg M.F. (2009). Determination of the plasticizer content in poly(vinyl acetate) paint medium by pyrolysis–silylation–gas chromatography–mass spectrometry. J. Anal. Appl. Pyrolysis.

[86] Hellgren A.-C., Weissenborn P., Holmberg K. (1999). Surfactants in water-borne paints. Prog. Org. Coat..

[87] Martens C.R. (1981). Waterborne Coatings: Emulsion and Water-Soluble Paints.

[88] Flick E.W. (1989). Handbook of Paint Raw Materials.

[89] Oldring P., Hayward G. (1987). Resins for Surface Coatings.

[90] Jablonski E., Learner T., Hayes J., Golden M. (2003). Conservation Concerns for Acrylic Emulsion Paints: A Literature Review. Tate Pap..

[91] França De Sá S., Viana C., Ferreira J.L. (2021). Tracing Poly(Vinyl Acetate) Emulsions by Infrared and Raman Spectroscopies: Identification of Spectral Markers. Polymers.

[92] Cappitelli F., Sorlini C. (2008). Microorganisms Attack Synthetic Polymers in Items Representing Our Cultural Heritage. Appl. Environ. Microbiol..

[93] Cappitelli F., Zanardini E., Sorlini C. (2004). The Biodeterioration of Synthetic Resins Used in Conservation. Macromol. Biosci..

[94] Amann M., Minge O. (2012). Biodegradability of Poly(vinyl acetate) and Related Polymers. Adv. Polym. Sci..

[95] Aruldass S., Mathivanan V., Mohamed A.R., Tye C.T. (2019). Factors affecting hydrolysis of polyvinyl acetate to polyvinyl alcohol. J. Environ. Chem. Eng..

[96] Wheeler O.L., Ernst S.L., Crozier R.N. (1952). Molecular weight degradation of polyvinyl acetate on hydrolysis. J. Polym. Sci..

[97] Doménech-Carbó M.T., Bitossi G., de la Cruz-Cañizares J., Bolívar-Galiano F., López-Miras M.d.M., Romero-Noguera J., Martín-Sánchez I. (2009). Microbial deterioration of Mowilith DMC 2, Mowilith DM5 and Conrayt poly(vinyl acetate) emulsions used as binding media of paintings by pyrolysis-silylation-gas chromatography–mass spectrometry. J. Anal. Appl. Pyrolysis.

[98] Bradford E.B., Vanderhoff J.W. (1972). Additional studies of morphological changes in latex films. J. Macromol. Sci. Part B.

[99] Scott N.D., Bristol J.E. Hydrolysis of Polymerized Vinyl Esters. https://patents.google.com/patent/US2266996A/en.

[100] Marin E., Rojas J., Ciro Y. (2014). A review of polyvinyl alcohol derivatives: Promising materials for pharmaceutical and biomedical applications. Afr. J. Pharm. Pharmacol..

[101] Clarke J.T., Blout E.R. (1946). Nature of the carbonyl groups in polyvinyl alcohol. J. Polym. Sci..

[102] Ferreira J.L., Melo M.J., Ramos A.M. (2006). A mural painting by Estrela Faria: A FTIR study of a vinyl synthetic medium. Contribution of the Seventh Biennial Gathering of the Infrared and Raman User’s Group.

[103] Melo M.J., Ferreira J.L., Ramos A.M., Avila M. (2006). Vinyl paints in Portugese modern art (1960–1990): A FTIR study. Contributions of the Sevents Biennial Gathering of the Infrared and Raman User’s Group.

[104] Ormsby B., Learner T. (2009). The effects of wet surface cleaning treatments on acrylic emulsion artists’ paints—A review of recent scientific research. Stud. Conserv..

[105] Erlebacher J., Browne E., Tumosa C., Mecklenburg M.F. (1992). The Effects of Temperature and Relative Humidity on the Rapidly Loaded Mechanical Properties of Artists’ Acrylic Paint. Materials Issues in Art and Archaeology III.

[106] Erlebacher J.D., Mecklenburg M.F., Tumosa C.S. (1992). The Mechanical Behavior of Artist’s Acrylic Paints with Changing Temperature and Relative Humidity. Polym. Prepr..

[107] Hagan E., Murray A. (2005). Effects of Water Exposure on the Mechanical Properties of Early Artists’ Acrylic Paints. MRS Proceedings.

[108] dePolo G., Walton M., Keune K., Shull K.R. (2021). After the paint has dried: A review of testing techniques for studying the mechanical properties of artists’ paint. Herit. Sci..

[109] Dillon C.E., Lagalante A.F., Wolbers R.C. (2014). Acrylic emulsion paint films: The effect of solution pH, conductivity, and ionic strength on film swelling and surfactant removal. Stud. Conserv..

[110] del Gaudio I., Hunter-Sellars E., Parkin I.P., Williams D., Da Ros S., Curran K. (2021). Water sorption and diffusion in cellulose acetate: The effect of plasticisers. Carbohydr. Polym..

[111] David C., Borsu M., Geuskens G. (1970). Photolysis and radiolysis of polyvinyl acetate. Eur. Polym. J..

[112] Geuskens G., Borsu M., David C. (1972). Photolysis and radiolysis of polyvinylacetate—II. Eur. Polym. J..

[113] Buchanan K.J., McGill W.J. (1980). Photodegradation of poly(vinyl esters)—I. Formation and quantitative measurement of volatile products. Eur. Polym. J..

[114] Buchanan K.J., McGill W.J. (1980). Photodegradation of poly(vinyl esters)—II. Volatile product formation and changes in the absorption spectra and molecular mass distributions. Eur. Polym. J..

[115] Buchanan K.J., McGill W.J. (1980). Photodegradation of poly(vinyl esters)—III. Photolysis mechanism for both polymeric and low molecular mass esters. Eur. Polym. J..

[116] Vaidergorin E.Y.L., Marcondes M.E.R., Toscano V.G. (1987). Photodegradation of poly(vinyl acetate). Polym. Degrad. Stab..

[117] Madras G., Chattopadhyay S. (2001). Optimum temperature for oxidative degradation of poly(vinyl acetate) in solution. Chem. Eng. Sci..

[118] Norrish R.G.W., Bamford C.H. (1937). Photo-decomposition of aldehydes and ketones. Nature.

[119] Wei S., Pintus V., Schreiner M. (2012). Photochemical degradation study of polyvinyl acetate paints used in artworks by Py–GC/MS. J. Anal. Appl. Pyrolysis.

[120] Melchiorre Di Crescenzo M., Zendri E., Sánchez-Pons M., Fuster-López L., Yusá-Marco D.J. (2014). The use of waterborne paints in contemporary murals: Comparing the stability of vinyl, acrylic and styrene-acrylic formulations to outdoor weathering conditions. Polym. Degrad. Stab..

[121] Pintus V., Wei S., Schreiner M. (2016). Accelerated UV ageing studies of acrylic, alkyd, and polyvinyl acetate paints: Influence of inorganic pigments. Microchem. J..

[122] Doménech-Carbó M.T., Silva M.F., Aura-Castro E., Fuster-López L., Kröner S., Martínez-Bazán M.L., Más-Barberá X., Mecklenburg M.F., Osete-Cortina L., Doménech A. (2011). Study of behaviour on simulated daylight ageing of artists’ acrylic and poly(vinyl acetate) paint films. Anal. Bioanal. Chem..

[123] Toja F., Saviello D., Nevin A., Comelli D., Lazzari M., Valentini G., Toniolo L. (2013). The degradation of poly(vinyl acetate) as a material for design objects: A multi-analytical study of the Cocoon lamps. Part 2. Polym. Degrad. Stab..

[124] Naude K.M., Styler S.A., Ormsby B. Photochemical Degradation of Non-Ionic Paint Surfactants: Implications for Art Conservation.

[125] Holland B.J., Hay J.N. (2002). The thermal degradation of poly(vinyl acetate) measured by thermal analysis–Fourier transform infrared spectroscopy. Polymer.

[126] Grassie N. (1952). The thermal degradation of polyvinyl acetate. Part 1. Products and reaction mechanism at low temperatures. Trans. Faraday Soc..

[127] Grassie N. (1953). The thermal degradation of polyvinyl acetate. Part 2. Determination of the rate constants of the primary processes involved in the elimination of acetic acid. Trans. Faraday Soc..

[128] Servotte A., Desreux V. (1968). Thermal degradation of some vinyl polymers. I. Poly(vinyl acetate). J. Polym. Sci. Part C Polym. Symp..

[129] Bataille P., Van B.T. (1975). Mechanism of thermal degradation of poly(vinyl acetate). J. Therm. Anal..

[130] Ballistreri A., Foti S., Montaudo G., Scamporrino E. (1980). Evolution of aromatic compounds in Tthe thermal decomposition of vinyl polymers. J. Polym. Sci. Part A-1 Polym. Chem..

[131] Troitskii B.B., Razuvaev G.A., Khokhlova L.V., Bortnikov G.N. (1973). On the mechanism of the thermal degradation of polyvinyl acetate. J. Polym. Sci. Polym. Symp..

[132] Rimez B., Rahier H., Van Assche G., Artoos T., Biesemans M., Van Mele B. (2008). The thermal degradation of poly(vinyl acetate) and poly(ethylene-co-vinyl acetate), Part I: Experimental study of the degradation mechanism. Polym. Degrad. Stab..

[133] Rimez B., Rahier H., Van Assche G., Artoos T., Van Mele B. (2008). The thermal degradation of poly(vinyl acetate) and poly(ethylene-co-vinyl acetate), Part II: Modelling the degradation kinetics. Polym. Degrad. Stab..

[134] Izzo F.C., Balliana E., Pinton F., Zendri E. (2014). A preliminary study of the composition of commercial oil, acrylic and vinyl paints and their behaviour after accelerated ageing conditions. Conserv. Sci. Cult. Herit..

[135] Pintus V., Viana C., Angelin E.M., De Sá S.F., Wienland K., Sterflinger K., Ferreira J.L. (2022). Applicability of single-shot and double-shot Py-GC/MS for the detection of components in vinyl acetate-based emulsions used in modern-contemporary art. J. Anal. Appl. Pyrolysis.

[136] Schossler P., Fortes I., Júnior J.C.D.d.F., Carazza F., Souza L.A.C. (2013). Acrylic and Vinyl Resins Identification by Pyrolysis-Gas Chromatography/Mass Spectrometry: A Study of Cases in Modern Art Conservation. Anal. Lett..

[137] Scalarone D., Chiantore O. (2009). Py-GC/MS of Natural and Synthetic Resins. Organic Mass Spectrometry in Art and Archaeology.

[138] Socrates G. (2010). Infrared and Raman Characteristic Group Frequencies: Tables and Charts.

[139] Alderson S., Down J.L., Maines C.A., Williams R.S., Young G.S. (2019). Potential substitutes for discontinued poly(vinyl acetate) resins used in conservation. J. Am. Inst. Conserv..

[140] Ormsby B., Learner T., Foster G., Druzik J., Schilling M. (2006). Wet cleaning acrylic emulsion paint films: An evaluation of physical, chemical, and optical changes. Modern Paints Uncovered: Proceedings from the Modern Paints Uncovered Symposium.

[141] Colombini M.P., Modugno F. (2009). Organic Mass Spectrometry in Art and Archaeology.

[142] Ropret P., Centeno S.A., Bukovec P. (2008). Raman identification of yellow synthetic organic pigments in modern and contemporary paintings: Reference spectra and case studies. Spectrochim. Acta Part A Mol. Biomol. Spectrosc..

[143] Fremout W., Saverwyns S. (2012). Identification of synthetic organic pigments: The role of a comprehensive digital Raman spectral library. J. Raman Spectrosc..

[144] Jovanović V., Erić S., Colomban P., Kremenović A. (2019). Identification of Lithol Red Synthetic Organic Pigment Reveals the Cause of Paint Layer Degradation on the Lazar Vozarević Painting “Untitled” with Copper Plates. Heritage.

[145] Scherrer N.C., Stefan Z., Francoise D., Annette F., Renate K. (2009). Synthetic organic pigments of the 20th and 21st century relevant to artist’s paints: Raman spectra reference collection. Spectrochim. Acta Part A Mol. Biomol. Spectrosc..

[146] Vandenabeele P., Edwards H.G.M., Moens L. (2007). A Decade of Raman Spectroscopy in Art and Archaeology. Chem. Rev..

[147] Kehlet C., Nunberg S., Alcala S., Dittmer J. (2018). Nuclear magnetic resonance analysis for treatment decisions: The case of a white sculptural environment by Louise Nevelson. Microchem. J..

[148] Souza C., Tavares M.I. (1998). Nuclear magnetic resonance study of commercial poly(vinyl acetate). J. Appl. Polym. Sci..

[149] Learner T. (2001). The analysis of synthetic paints by pyrolysis-gas chromatography-mass spectrometry (PyGCMS). Stud. Conserv..

[150] Peris-Vicente J., Baumer U., Stege H., Lutzenberger K., Gimeno Adelantado J.V. (2009). Characterization of Commercial Synthetic Resins by Pyrolysis-Gas Chromatography/Mass Spectrometry: Application to Modern Art and Conservation. Anal. Chem..

[151] Ormsby B., Learner T., Schilling M., Druzik J., Khanjian H., Carson D., Foster G., Sloan M. The Effects of Surface Cleaning on Acrylic Emulsion Paintings: A Preliminary Investigation. Tate Papers No 6. https://www.tate.org.uk/research/tate-papers/06/effects-of-surface-cleaning-on-acrylic-emulsion-painting-preliminary-investigation.

[152] Lee J., Bonaduce I., Modugno F., La Nasa J., Ormsby B., van den Berg K.J. (2018). Scientific investigation into the water sensitivity of twentieth century oil paints. Microchem. J..

[153] Fardi T., Pintus V., Kampasakali E., Pavlidou E., Papaspyropoulos K.G., Schreiner M., Kyriacou G. (2018). A novel methodological approach for the assessment of surface cleaning of acrylic emulsion paints. Microchem. J..

[154] Smithen P. (2006). A history of the treatment of acrylic painting. Modern Paints Uncovered.

[155] Roy P., Hackney S., Townsend J., Eastaugh N. (1990). Problems of dirt accumulation and its removal from unvarnished paintings: A practical review. Dirt and Pictures Separated.

[156] Murray A., de Berenfeld C.C., Chang S.Y.S., Jablonski E., Klein T., Riggs M.C., Robertson E.C., Tse W.M.A. (2002). The Condition and Cleaning of Acrylic Emulsion Paintings. MRS Online Proc. Libr..

[157] Cardaba I., Poggi G., Baglioni M., Chelazzi D., Maguregui I., Giorgi R. (2020). Assessment of aqueous cleaning of acrylic paints using innovative cryogels. Microchem. J..

[158] Angelova L.V., Ormsby B., Richardson E. (2016). Diffusion of water from a range of conservation treatment gels into paint films studied by unilateral NMR: Part I: Acrylic emulsion paint. Microchem. J..

[159] Ormsby B., Learner T. (2014). Artists’ acrylic emulsion paints: Materials, meaning and conservation treatment options. AICCM Bull..

[160] Kampasakali E., Ormsby B., Cosentino A., Miliani C., Learner T. (2011). A Preliminary Evaluation of the Surfaces of Acrylic Emulsion Paint Films and the Effects of Wet-Cleaning Treatment by Atomic Force Microscopy (AFM). Hastings Cent. Stud..

[161] Chelazzi D., Fratini E., Giorgi R., Mastrangelo R., Rossi M., Baglioni P. (2018). Gels for the Cleaning of Works of Art. ACS Symposium Series.

[162] Angelova L.V., Ormsby B., Townsend J., Wolbers R., International Academic Projects, Tate Modern (Gallery) (2017). Gels in the Conservation of Art.

[163] Baglioni P., Berti D., Bonini M., Carretti E., Dei L., Fratini E., Giorgi R. (2013). Micelle, microemulsions, and gels for the conservation of cultural heritage. Adv. Colloid Interface Sci..

[164] Stoveland L.P., Frøysaker T., Stols-Witlox M., Grøntoft T., Steindal C.C., Madden O., Ormsby B. (2021). Evaluation of novel cleaning systems on mock-ups of unvarnished oil paint and chalk-glue ground within the Munch Aula Paintings Project. Herit. Sci..

[165] Chung J.Y., Ormsby B., Lee J., Burnstock A., Bridgland J. (2017). An investigation of options for surface cleaning unvarnished water-sensitive oil paints based on recent developments for acrylic paints. ICOM-CC 18th Triennial Conference Preprints.

[166] Ormsby B., Kampasakali E., Learner T. (2013). Surfactants and acrylic dispersion paints: Evaluating changes induced by wet surface cleaning treatments. Cleaning 2010 Congress New Insights into the Cleaning of Paintings.

[167] Hagan E.W.S., Charalambides M.N., Young C.T., Learner T.J.S., Hackney S. (2009). Tensile properties of latex paint films with TiO_2_ pigment. Mech. Time-Depend. Mater..

[168] Meek M.E., Long G. (1997). Di-n-Butyl Phthalate.

[169] Staples C.A., Peterson D.R., Parkerton T.F., Adams W.J. (1997). The environmental fate of phthalate esters: A literature review. Chemosphere.

[170] Sekizawa J., Dobson S. (2003). Diethyl Phthalate.

[171] Lundberg P. (1992). Diethylhexyl Phthalate.

[172] (2014). Diisobutyl Phthalate (DIBP). https://echa.europa.eu/documents/10162/12934bbe-ad6f-4671-a931-1ef44f51cb10.

[173] Diisobutyl Phthalate—The Chemical Company. https://thechemco.com/chemical/diisobutyl-phthalate/.

[174] Programme International sur la Sécurité des Substances Chimiques (1991). Triphenyl Phosphate.

[175] (2012). Polyethylene glycol [MAK Value Documentation, 1998]. The MAK-Collection for Occupational Health and Safety.

